# The effects of betaine supplementation on fluid balance and heat tolerance during passive heat stress in men

**DOI:** 10.14814/phy2.15792

**Published:** 2023-08-21

**Authors:** Brandon D. Willingham, Liliana I. Rentería, Tristan J. Ragland, Michael J. Ormsbee

**Affiliations:** ^1^ Department of Kinesiology Coastal Carolina University Conway South Carolina USA; ^2^ Institute of Sports Sciences & Medicine, Nutrition, and Integrative Physiology Florida State University Florida USA; ^3^ Department of Kinesiology and Health Rutgers University New Brunswick New Jersey USA; ^4^ Discipline of Biokinetics, Exercise, and Leisure Sciences University of KwaZulu‐Natal Durban South Africa

**Keywords:** betaine, core temperature, fluid balance, heat stress, hydration, plasma volume

## Abstract

**Introduction:**

Consuming intracellular osmolytes, like betaine (BET), may attenuate symptoms of heat stress. The purpose of this study was to examine the effects of BET supplementation on fluid balance and heat tolerance after a 7‐day loading period and during passive heat exposure.

**Methods:**

A double‐blind, placebo controlled, crossover study compared BET or placebo consumption (50 mg·kg^−1^, twice daily) for 7 days in young, recreationally active men (N = 11).

**Results:**

During the loading period, no significant interactions were found for any marker of fluid balance between or within conditions. During heat exposure, significant time effects but no condition x time interactions, were found for plasma characteristics (i.e., volume, osmolality, sodium, albumin, and total protein). Plasma volume was significantly increased by min 30 in both conditions (PLA: +6.9. ± 5.0%, BET: +10.2 ± 7.4%) and remained elevated for the remainder of the experimental trial, but was not significantly different between conditions. After 60 min of passive heat exposure, both conditions experienced a similar increase in core temperature (PLA: +0.32 ± 0.22°C, BET: +0.31 ± 0.21°C; *p* = 0.912).

**Conclusions:**

Supplemental BET did not improve markers of fluid balance or heat tolerance during 7 days of loading or during passive heat exposure.

## 1 INTRODUCTION

Heat stress increases all‐cause mortality, and is responsible for the death of thousands globally (Argaud et al., 2007; Azhar et al., 2014; Hoffmann et al., 2008), including approximately 620 deaths in the United States each year (Berko et al., 2014). Thermal stress is known to challenge thermoregulation, fluid balance, and gut permeability increasing the risk of heat related injury if unmitigated (Nichols, 2014; Périard et al., 2021). As climates change and the world becomes hotter, it is critical to investigate physiological interventions that may potentially mitigate these catastrophic events. One such, under‐explored, nutritional strategy is the consumption of trimethylglycine, henceforth referred to as betaine (BET) (Willingham et al., 2020).

BET may improve acute heat tolerance both as an intracellular osmolyte and molecular chaperone. As an osmolyte, BET may increase intracellular fluid volume and minimize cellular hypertonic stress. Indeed, mammalian cells combat hypertonic stress by accumulating BET by upregulating the BET transporter (Alfieri et al., 2002; Sheikh‐Hamad et al., 1994). Through its role as a molecular chaperone, BET can refold denatured proteins or stabilize proper conformation of proteins that may otherwise become denatured when facing thermal or hypertonic stress (Bruzdźiak et al., 2016; Fan et al., 2012; Seeliger et al., 2013). Importantly, it appears that BET is more effective at preventing the initial denaturation process compared to refolding (Bruzdźiak et al., 2016); highlighting the need for BET to be present before the stressor is applied. If an exogenous molecular chaperone, such as BET, is adequately protecting the cellular environment during heat stress, then the need for endogenous molecular chaperones (i.e., heat shock proteins; HSP) would be reduced. Indeed, this has been demonstrated in cell culture (Alfieri et al., 2004; Sheikh‐Hamad et al., 1994) and animal models (Dangi et al., 2016), but there are no human studies examining BET's role in HSP expression to thermal stress.

Furthermore, heat related injuries are partly attributed to endotoxemia, the presence of gut bacteria in the blood (Snipe et al., 2018; Vargas & Marino, 2016). During passive heat stress, functional sympatholysis shunts blood flow away from the viscera, reducing intestinal blood flow by 30%–60% in humans (Rowell et al., 1969; Rowell et al., 1971) and animals alike (Hales et al., 1979; Wolfenson et al., 1981). Collectively, the combination of decreased blood flow, alongside increased thermal and hypertonic stress, exacerbate intestinal permeability (Matthes et al., 2017; Walsh et al., 2011). Lipopolysaccharide (LPS) is known to translocate from the enterocytes into circulation when the gastrointestinal (GI) tract becomes compromised leading to endotoxemia (Pires et al., 2017). BET may defend the integrity of the enterocytes against endotoxemia by mitigating the thermal and hypertonic stress, thus reducing plasma LPS during heat stress. Indeed, while there is no human data, BET has been shown to improve gut health via increased intestinal villi height, villi area, crypt depth, and transepithelial resistance in chickens undergoing passive heat stress (Shakeri et al., 2020).

In fact, most of the research examining BET's role in thermal stress has been performed in cell culture and animal models experiencing passive heat stress. Collectively, these data tend to show evidence of reduced thermal stress when exposed to passive heat (DiGiacomo et al., 2016; Egbuniwe et al., 2018; Khattak et al., 2012; Ratriyanto & Mosenthin, 2018; Sahebi Ala et al., 2019; Shakeri et al., 2018; Zhang et al., 2014). Notably, improved heat tolerance is observed when BET is introduced chronically into the feed or water supply (DiGiacomo et al., 2016; Egbuniwe et al., 2018; Khattak et al., 2012; Ratriyanto & Mosenthin, 2018; Sahebi Ala et al., 2019; Shakeri et al., 2018; Zhang et al., 2014). Despite these positive findings in animal models, there is a gap of understanding regarding BET's ability to attenuate thermal stress in humans in either passive or active heat stress scenarios. Only one published study has specifically examined thermal stress in response to BET in humans (Armstrong et al., 2008). This study investigated the acute use of BET in rehydration after a dehydrating protocol in the heat (31.1 ± 0.7°C, 34.7 ± 5.5% relative humidity [RH]). Specifically, Armstrong et al. examined a single dose of BET (5 g dissolved in 1 L of water) as a rehydration solution, thereby introducing BET after the initial thermal stress was applied. In doing so, BET's ability to provide cellular defense in the midst of thermal stressors was effectively negated (Bruzdźiak et al., 2016; Fan et al., 2012). Therefore, it is no surprise that these authors report limited data supporting BET's ability to combat thermal stress. We propose BET supplementation should be pre‐loaded, mimicking the successful animal models. Therefore, the purpose of this study was to examine the effects of preloaded BET supplementation (50 mg·kg^−1^, twice daily for 7 days) on fluid balance and heat tolerance in recreationally active young men undergoing passive heat stress. We hypothesize that BET supplementation will improve markers of fluid balance and heat tolerance as evidenced by (1) increased ICF after the loading period and (2) an attenuated rise in core temperature, plasma LPS, and serum HSP70.

## 2 METHODS

### 2.1 Design

**FIGURE 1:**
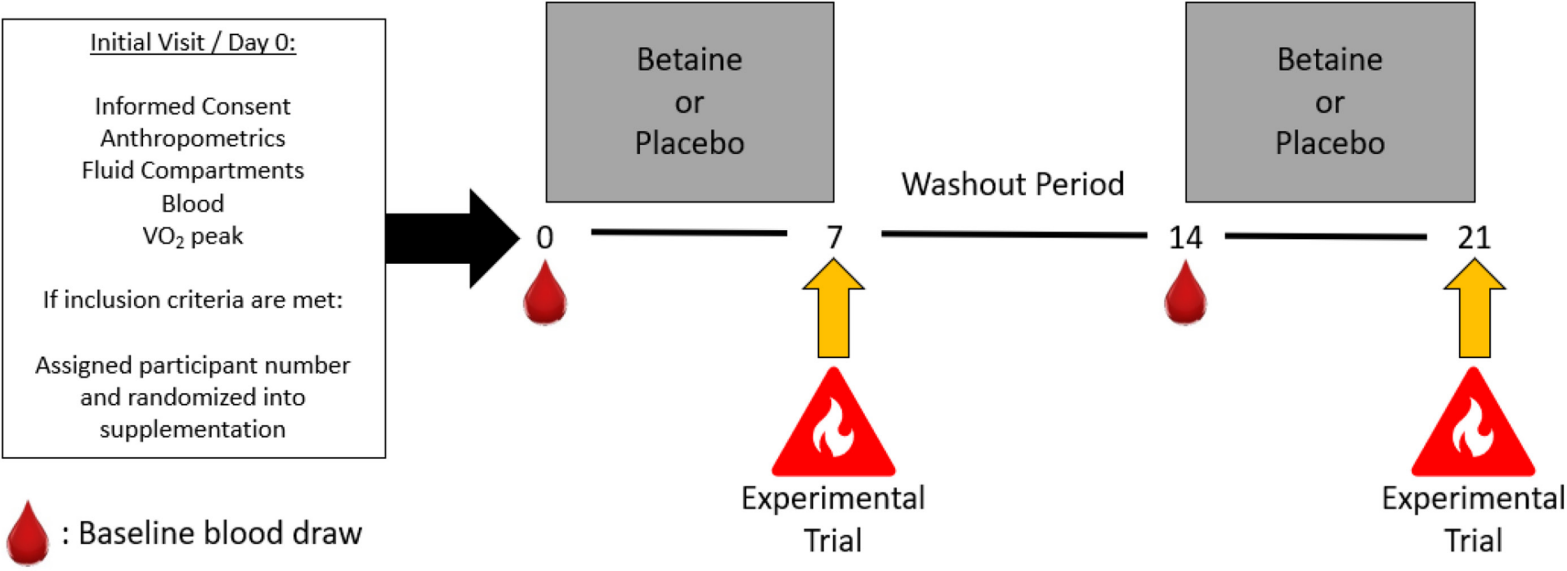
Study outline.

This was a double blind, placebo‐controlled, randomized, crossover study. After completing an initial visit (described below), participants were randomly assigned to either BET or a noncaloric placebo (PLA) supplemental condition. Participants consumed 50 mg·kg^−1^ (rounded up to the nearest gram) of BET (NOW Foods, Bloomingdale, IL) or PLA (noncaloric rice flour), twice daily (~12 h apart) with 6 mL·kg^−1^ water with each dose. Supplementation occurred from days 0–6 and 14–20, with the last dosage consumed ~12 h prior to the two experimental visits on day 7 and day 21. On day 7, participants completed the first experimental trial involving exposure to uncompensible passive heat stress (40°C, 60% RH) (Vecellio et al., 2022) in an environmental chamber for 60 min, followed by a 30 min thermoneutral recovery period outside of the chamber (23°C, 50% RH). Afterwards, participants entered a 7 day washout period (Schwahn et al., 2003) before crossing over to the second supplemental condition on day 14. On day 21, after 7 more days of supplementation, participants completed their second experimental trial. To ensure participants arrived in a similar physiological status, participants recorded and replicated their diet (24 h) and training (7 days) prior to each experimental visit. Oral and written informed consent were attained prior to participation. The study design (Figure 1) was approved by the Florida State University Institutional Review Board (IRB # 00000259).

### 2.2 Standardization of diet and training

To standardize baseline plasma BET concentration, participants were asked to eliminate food sources that contain the highest concentrations of BET from their diet throughout the duration of the study (e.g., wheat products, spinach, beets, pretzels, and shrimp) (Zeisel et al., 2003). Prior to each experimental visit, participants were asked to maintain consistent dietary and exercise habits for 24 h, which was confirmed with a 24 h food and exercise log and replicated before the second experimental visit.

### 2.3 Initial visit

For every laboratory visit, participants arrived in the morning, following an overnight fast (7–9 h) having abstained from caffeine (previous 12 h), alcohol, and exercise (previous 24 h). For the initial visit, participants were asked to consume 16 ounces of water 60 min prior to arrival. After obtaining written and verbal consent, participants' height (Seca), weight to the nearest 0.1 kg (Detecto® 750, Webb City, MO), and body composition (Bod Pod, Cosmed) were assessed. Thereafter, participants rested quietly in a supine position for 5 min, before fluid compartment volumes were measured via bioelectric impedance spectroscopy (BIS; ImpediMed SFB7, ImpediMed Limited) (Moon, 2013). Fluid compartment volumes were assessed three times in succession and the average of the three measures was recorded and used in the analysis. Then, in a seated position, blood was collected to establish pre‐supplementation baseline data. Following these baseline measurements, participants were asked to complete the YMCA submaximal cycling protocol for the assessment of cardiorespiratory fitness. This test, instead of a maximal test, was chosen to satisfy the COVID‐19 safety protocols at the time of data collection. Participants were then provided with their blinded supplement and instructed to consume 50 mg·kg^−1^ twice daily (~12 h apart) with 6 mL·kg^−1^ water with each dose, for 7 days (Schwahn et al., 2003).

### 2.4 Experimental trials

**FIGURE 2:**
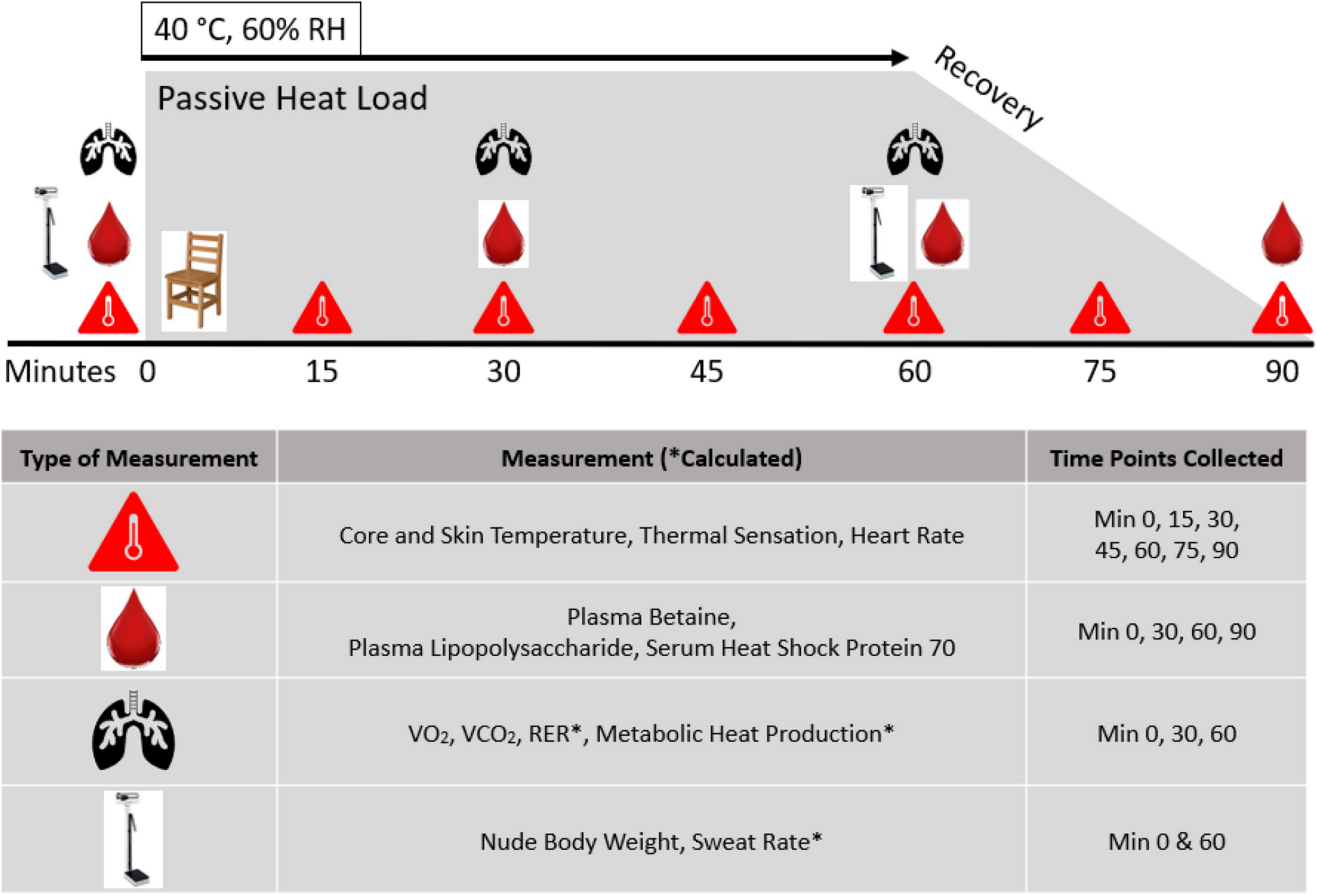
Experimental trials.

Participants ingested a CorTemp Sensor (HQInc, Palmetto, FL) the evening prior to experimental visits, 10–12 h before arriving to the laboratory. Likewise, participants consumed 6 mL·kg^−1^ water 60 min prior to arrival and were otherwise fasted. Upon arrival to the experimental trials (Figure 2), participants provided a urine sample to confirm euhydration (USG <1.020) (Armstrong et al., 1994; Cheuvront et al., 2015; Pross et al., 2013). While USG was used as the immediate indicator of hydration, plasma sodium and osmolality data were used to confirm these findings. After nude body mass (BM) was recorded, participants rested quietly in a supine position for 5 min before fluid compartment volumes were assessed via BIS (Moon, 2013). Participants were then fitted with iButtons (iButton DS1921G‐F5, Maxim Integrated) to estimate whole body skin temperature from nine standardized locations on the body (Choi et al., 1997). Mean skin temperature was calculated based on the modified Gagge and Nishi formula which has been validated at high ambient temperatures (28°C and 40°C) similar to those found in this study (40°C) (Choi et al., 1997). The modified Gagge and Nishi Formula (Choi et al., 1997; Gagge & Nishi, 2010) is as follows:

$$\begin{array}{c} \text{Mean Skin Temperature}\\ \;=\left(T_{\text{CHEEK}}\times 0.07\right)+\left(T_{\text{ABDOMEN}}\times 0.175\right)\\ \;+\left(T_{\text{SUBSCAPULA}}\times 0.175\right)+\left(T_{\text{UPPERARM}}\times 0.07\right)\\ \;+\left(T_{\text{FOREARM}}\times 0.07\right)+\left(T_{\text{HAND}}\times 0.05\right)\\ \;+\left(T_{\text{THIGH}}\times 0.19\right)+\left(T_{\text{CALF}}\times 0.13\right)\\ \;+\left(T_{\text{FOOT}}\times 0.07\right) \end{array}$$

Participants were then seated in an upright position and fitted with a heart rate monitor (Polar® FT4M). Baseline measures of core temperature, skin temperature, heart rate, thermal sensation and subjective ratings of thirst were recorded. Thermal sensation was assessed using a 9‐point Likert scale, ranging from −4 (Unbearably Cold), to +4 (Unbearably Hot) with 0 (Comfortable) anchored at the midpoint. Perceived thirst was measured using a 100 mm long psychometric response scale with verbal anchors at assigned distances to quantify subjective measures. Thereafter, the first blood sample was drawn from the antecubital space. Participants did not eat or drink until all experimental data collection was completed.

Following baseline measurements, participants entered the environmental chamber (40°C, 60% RH) to begin 60 min of seated passive heat exposure. Participants wore a standardized outfit (i.e., t‐shirt, shorts, socks, and shoes) while in the chamber for every experimental condition. Once inside, measures of core temperature, skin temperature, thermal sensation, and heart rate were measured in 15‐min intervals. Blood was drawn inside the chamber at min 30 and 60. Breath measurements were collected from 0 to5 min, 25 to 30 min, and 55 to 60 min. Metabolic heat production was calculated using measures of gas exchange (i.e., VO_2_ and RER) as well as the known caloric equivalents of CHO (21.12 kJ·L^−1^ O_2_ consumed) and fat (19.61 kJ·L^−1^ O_2_ consumed) (Cramer & Jay, 2016; Murgatroyd et al., 1993). The calculation for metabolic heat production is as follows:

$$\begin{array}{c} \begin{array}{c} \text{Metabolic Heat Production}={\mathrm{VO}}_{2}\cdot \frac{\left(\left(\frac{\mathrm{RER}-0.7}{0.3}\right)\;\mathrm{EC}\right)+\left(\left(\frac{1.0-\mathrm{RER}}{0.3}\right)\;\mathrm{EF}\right)}{60}*1000\;\left(W\right) \end{array} \end{array}$$

After 60 min of passive heat exposure, participants exited the chamber. Immediately afterward, participants completed their subjective measure of thirst, then toweled dry to provide their post‐heat exposure nude body weight. Thereafter, participants were seated and passively recovered in a thermoneutral room (23°C, 50% RH) for 30 min before the final blood draw.

### 2.5 Analysis of blood

During the initial visits (at baseline assessment) and experimental visits (min 0, 30, 60, and 90), venous blood was collected from the antecubital forearm vein by trained phlebotomists under sterile conditions. Per time point, blood was drawn into vacutainer tubes coated in K_2_EDTA (BD Vacutainer®, Franklin Lakes, NJ), vacutainer tubes coated in lithium heparin (VWR, Atlanta, GA), and into serum vacutainer tubes (VWR, Atlanta, GA). Thereafter, the blood from the K_2_EDTA and lithium heparin tubes were centrifuged for 15 min at 3500 rpm at 4°C to acquire plasma. Plasma was then separated into aliquots and stored at −80°C for later batch analysis.

Whole blood drawn into the serum vacutainer was immediately separated into micro‐hematocrit capillary tubes (Thomas Scientific) and analyzed for hemoglobin concentration (OSM‐3, Radiometer) and, after centrifugation, hematocrit percentage. The remaining blood from the serum vacutainer tube was allowed to clot at room temperature for 30 min, then centrifuged for 15 min at 3500 rpm at 4°C. Serum was then separated into aliquots and stored at −80°C for later batch analysis.

Change in plasma volume was calculated using the Dill and Costill method (Dill & Costill, 1974). Whereas, plasma electrolytes (Na^+^, K^+^, Cl^−^), proteins (albumin and total plasma proteins), glucose, and blood urea nitrogen (BUN) were measured using the Piccolo Xpress Chemistry Analyzer (Abaxis Inc). Plasma BET was analyzed via high performance liquid chromatography as described elsewhere (Laryea et al., 1998). Plasma osmolality was calculated using the Worthley equation below (Worthley et al., 1987).

$$\begin{array}{c} \begin{array}{c} \;\text{Plasma Osmolality}\;\left(\text{mOsm}/\mathrm{kg}\right)=\left[2\times \left[\mathrm{Na}\;\left(\text{mmol}/L\right)\right]\right]+\frac{\left[\text{Glucose (mg/dL)}\right]}{18}+\frac{\left[\text{BUN (mg/dL)}\right]}{2.8} \end{array} \end{array}$$

### 2.6 Statistics

In accordance with the specific aims, two a priori power analyses (JMP Pro 12) for primary physiological outcomes were conducted.

To date, no significant differences in core temperature have been observed using BET in human trials. Therefore, the power analysis for core temperature was determined using a non‐ human, BET model exposing sheep to a passive heat load (43°C, 49% RH) (DiGiacomo et al., 2016), where there was a 0.2°C mean difference between hot and neutral conditions, and an observed standard deviation of 0.064°C for the control group at 90% power. It was determined, using JMP Pro, that a sample size of 7 would be required to detect significant differences in core temperature.

Similarly, the power calculation for change in plasma volume is based upon a BET supplementation study in a human model reporting a mean difference of 3.3% pre‐ versus post‐ 36 min of exercise (running at 65% VO_2_ peak for 75 min), and a mean standard deviation of 1.1% at 90% power (Armstrong et al., 2008). It was determined that a sample size of 8 would be required to detect significant differences in plasma volume changes. Using the most conservative value found (N = 8), and accounting for a ~ 33% attrition rate, we recruited 11 participants.

All variables were assessed using null‐hypothesis testing, with significance set at *p* ≤ 0.05. Analyses were conducted using SPSS version 25.0 (SPSS, Inc), and data are presented as means ± SD.

To evaluate the impact of preloaded BET supplementation on fluid balance in recreationally active men, measures of fluid compartments (i.e., ICF, ECF, TBW, and plasma osmolality), plasma electrolytes (Na^+^, K^+^, Cl^−^) and proteins (albumin and total plasma proteins) were compared using paired‐samples t‐tests before and after 7‐days of supplementation.

To determine the degree to which preloading with BET impacts fluid balance and heat tolerance relative to placebo in young (ages 18–45 years), recreationally active men, paired‐samples t‐test and two‐way (treatment x time) repeated measures analysis of variance (RMANOVA) were used to identify differences. Specifically, sweat rate (i.e., changes in nude body weight) and subjective measures of thirst were compared at two time‐points (min 0 and 60) using paired‐samples *t*‐tests. Additionally, RMANOVA were used to determine if significant treatment, time, or treatment by time interactions exist for measures of fluid compartments (i.e., plasma osmolality and changes in plasma volume) as well as heat tolerance (i.e., serum HSP70 and Plasma LPS) between conditions across four time points (min 0, 30, 60, and 90). Measures of metabolism (i.e., VO_2_, respiratory exchange ratio [RER], and metabolic heat production) were assessed using RMANOVA across three time points (min 0, 30, and 60). Direct measures of heat tolerance (i.e., core temperature, skin temperature, heart rate, and subjective thermal sensation) were assessed using RMANOVA across 7 time points (min 0, 15, 30, 45, 60, 75, and 90).

If sphericity was violated, a Greenhouse–Geisser correction was performed. In the case of statistical significance, a post hoc one‐way ANOVA with Bonferroni tests were used to identify significant differences.

## 3 RESULTS

### 3.1 Participants

Eleven healthy, recreationally active men (age 29.1 ± 5.2 y; height 184.0 ± 7.8 cm; weight 78.5 ± 9.4 kg; body surface area 2.0 ± 0.2 m^2^; body fat 13.3 ± 6.9%, and estimated VO_2MAX_ 49.4 ± 17.0 mL·kg·min^−1^) participated in this study. All participants were free of any cardiovascular or metabolic conditions, had never experienced a prior heat related illness, and were not taking any supplements known to impact hydration or thermoregulatory responses. Based on the loading strategy (50 mg·kg^−1^, twice daily—approximately 12 h apart—for 7 days) and the participants' weights, average BET consumption was 8 g per day (4 g morning, 4 g evening), with two individuals receiving 9 g per day (4 g morning, 5 g evening). Each dose of BET was consumed with 6 mL·kg^−1^ water (averaging ~470 mL per dose) to closely match the only other study in humans examining BET impact on heat (Armstrong et al., 2008).

### 3.2 Loading betaine

The results for Aim 1 measuring changes in plasma BET concentrations, fluid compartments (i.e., ICF, ECF, TBW, and plasma osmolality), plasma electrolytes (Na^+^, K^+^, Cl^−^) and proteins (i.e., albumin and total plasma proteins) before and after the loading protocol are reported in Table 1. With the exception of plasma Na^+^, no significant differences were detected between conditions at baseline for any variable measured. As expected, after 7 days of loading, BET concentrations were significantly higher (*p* < 0.001) in BET compared to PLA. However, no significant differences were detected between BET and PLA for any other variable measured during this time. Interestingly, after 7 days of supplementation, BET tended to increase total plasma protein concentrations (Day 0: 7.16 ± 0.43 g·dL^−1^, Day 7: 7.47 ± 0.36 g·dL^−1^; *p* = 0.063), compared to PLA (Day 0: 7.20 ± 0.50 g·dL^−1^, Day 7: 7.35 ± 0.34 g·dL^−^1; *p* = 0.154).

**TABLE 1:** The effects of loading betaine on plasma characteristics of fluid balance.

Measure	Condition	Day 0	Day 7	Mean change	*p* Value
Plasma betaine (μmol·L^−1^)	PLA BET	39.7 ± 54.4 63.1 ± 63.3	21.4 ± 23.2 702.5 ± 233.7^a^	−18.3 +639.4	0.376 <0.001
ICF (L)	PLA BET	26.31 ± 4.05 26.21 ± 3.22	26.96 ± 3.26 26.62 ± 3.19	+0.65 +0.41	0.249 0.385
ECF (L)	PLA BET	20.02 ± 2.98 19.60 ± 2.14	20.02 ± 2.46 19.47 ± 1.99	+0.00 −0.13	0.992 0.493
TBW (L)	PLA BET	46.34 ± 6.80 45.81 ± 5.18	46.98 ± 5.61 46.09 ± 5.09	+0.64 +0.28	0.385 0.626
Plasma osmolality (mOsm·kg^−1^)	PLA BET	291.65 ± 5.97 295.58 ± 5.36	292.59 ± 6.68 294.29 ± 6.21	+0.94 −1.29	0.760 0.428
Na^+^(mmol·L^−1^)	PLA BET	140.64 ± 3.17 142.64 ± 2.73^a^	141.00 ± 2.93 141.73 ± 3.17	+0.36 −0.91	0.811 0.290
K^+^ (mmol·L^−1^)	PLA BET	4.41 ± 0.57 4.80 ± 0.47	4.74 ± 0.54 4.69 ± 0.49	+0.34 −0.11	0.076 0.524
Cl^−^ (mmol·L^−1^)	PLA BET	106.36 ± 2.87 107.50 ± 2.80	106.91 ± 2.30 105.50 ± 3.24	+0.55 −2.00	0.625 0.109
Albumin (g·dL^−1^)	PLA BET	4.11 ± 0.23 4.15 ± 0.22	4.22 ± 0.15 4.21 ± 0.16	+0.11 +0.06	0.126 0.418
Total protein (g·dL^−1^)	PLA BET	7.20 ± 0.50 7.16 ± 0.43	7.35 ± 0.34 7.47 ± 0.36	+0.15 +0.31	0.154 0.063

*Note*: *p*‐values indicate time effects (i.e., change from Day 0 to Day 7).

Abbreviations: BET, betaine, Cl^−^: chloride, ECF, extracellular fluid volume, ICF, intracellular fluid volume, K^+^, potassium, Na^+^, sodium, PLA, placebo, TBW, total body water.

^a^ Significantly different from PLA at the same time point (*p* < 0.05).

### 3.3 Betaine in the heat

The environmental conditions inside the chamber throughout passive heat exposure were 38.94 ± 0.35°C and 59.34 ± 3.27% RH. No significant differences were observed between conditions for temperature (*p* = 0.129) or humidity (*p* = 0.060), during the 60 min of passive heat stress. At the onset of the experimental visits, all participants were considered euhydrated as USG (PLA: 1.009 ± 0.008 g·dl^−1^, BET: 1.010 ± 0.007 g·dl^−1^; *p* = 0.794), plasma sodium (PLA: 141.00 ± 2.93 mmol·L^−1^, BET: 141.73 ± 3.17 mmol·L^−1^; *p* = 0.838) and plasma osmolality (PLA: 287.56 ± 5.99 mOsm·kg^−1^, BET: 289.16 ± 6.20 mOsm·kg^−1^; *p* = 0.513) were within appropriate ranges and not statistically different between conditions.

#### 3.3.1 Plasma betaine concentration

Supplementation significantly increased BET concentrations (Table 2) for all time points compared to PLA (*p* < 0.001). Yet, heat stress did not significantly change BET concentrations during the experimental visit (*p* = 0.455).

**TABLE 2:** Effects of betaine on blood characteristics during passive heat stress.

		Min 0	Min 30	Min 60	Min 90
Plasma BET (μmol·L^−1^)	PLA	21.4 ± 23.2	25.1 ± 30.1	30.7 ± 22.7	26.5 ± 18.9
	BET	702.5 ± 233.7^a^	707.0 ± 253.3^a^	743.0 ± 190.0^a^	722.7 ± 267.8^a^
Plasma volume (%, normalized to Min 0)	PLA	100 ± 0.0	106.9 ± 5.0*	104.4 ± 5.6*	104.3 ± 5.6*
	BET	100 ± 0.0	110.2 ± 7.4*	106.6 ± 6.6*	106.5 ± 4.8*
Plasma osmolality (mOsm·kg^−1^)	PLA	292.59 ± 6.68	288.97 ± 5.40*	290.62 ± 5.84	291.54 ± 4.83
	BET	294.29 ± 6.21	287.96 ± 6.48*	288.42 ± 6.65	292.30 ± 3.80
Na^+^ (mmol·L^−1^)	PLA	141.00 ± 2.93	139.30 ± 2.95*	140.10 ± 3.07	140.60 ± 2.37
	BET	141.73 ± 3.17	138.73 ± 3.29*	139.00 ± 3.13	140.82 ± 1.99
K^+^ (mmol·L^−1^)	PLA	4.74 ± 0.54	4.50 ± 0.25	4.50 ± 0.47	4.65 ± 0.58
	BET	4.69 ± 0.49	4.54 ± 0.33	4.54 ± 0.43	4.60 ± 0.30
Cl^−^ (mmol·L^−1^)	PLA	106.91 ± 2.30	106.60 ± 2.01	107.50 ± 1.96	105.90 ± 2.23
	BET	105.50 ± 3.24	107.19 ± 1.60	106.82 ± 1.83	106.27 ± 2.15
Albumin (g·dL^−1^)	PLA	4.22 ± 0.15	4.04 ± 0.14*	4.12 ± 0.15*	4.23 ± 0.21
	BET	4.21 ± 0.16	4.03 ± 0.13*	4.05 ± 0.12*	4.20 ± 0.15
Total proteins (g·dL^−1^)	PLA	7.35 ± 0.34	7.13 ± 0.41*	7.04 ± 0.87	7.35 ± 0.34
	BET	7.47 ± 0.36	7.09 ± 0.34*	7.19 ± 0.41	7.42 ± 0.40
Plasma LPS (pg·mL^−1^)	PLA	319.55 ± 115.73	212.37 ± 49.89	227.95 ± 76.24	201.25 ± 53.04
	BET	261.17 ± 116.47	212.92 ± 100.85	216.33 ± 103.56	199.52 ± 84.42
Serum HSP 70 (pg·mL^−1^)	PLA	70.88 ± 40.69	78.07 ± 42.07	84.04 ± 43.42	68.27 ± 35.75
	BET	81.36 ± 47.45	75.84 ± 34.60	77.52 ± 39.38	78.04 ± 48.20

*Note*: Min 0: Immediately prior to heat exposure, Min 30: 30 min of heat exposure, Min 60: 60 min of heat exposure, Min 90: 30 min of recovery in thermoneutral environment.

Abbreviations: BET, betaine, Cl^−^, chloride, HSP 70, heat shock protein 70, K^+^, potassium, LPS, lipopolysaccharide, Min, minute, Na^+^: sodium, PLA, placebo.

* Significantly different from Min 0 (*p* < 0.05).

^a^ Significantly different from PLA at the same time point (*p* < 0.05).

#### 3.3.2 Plasma volume and osmolality

**FIGURE 3:**
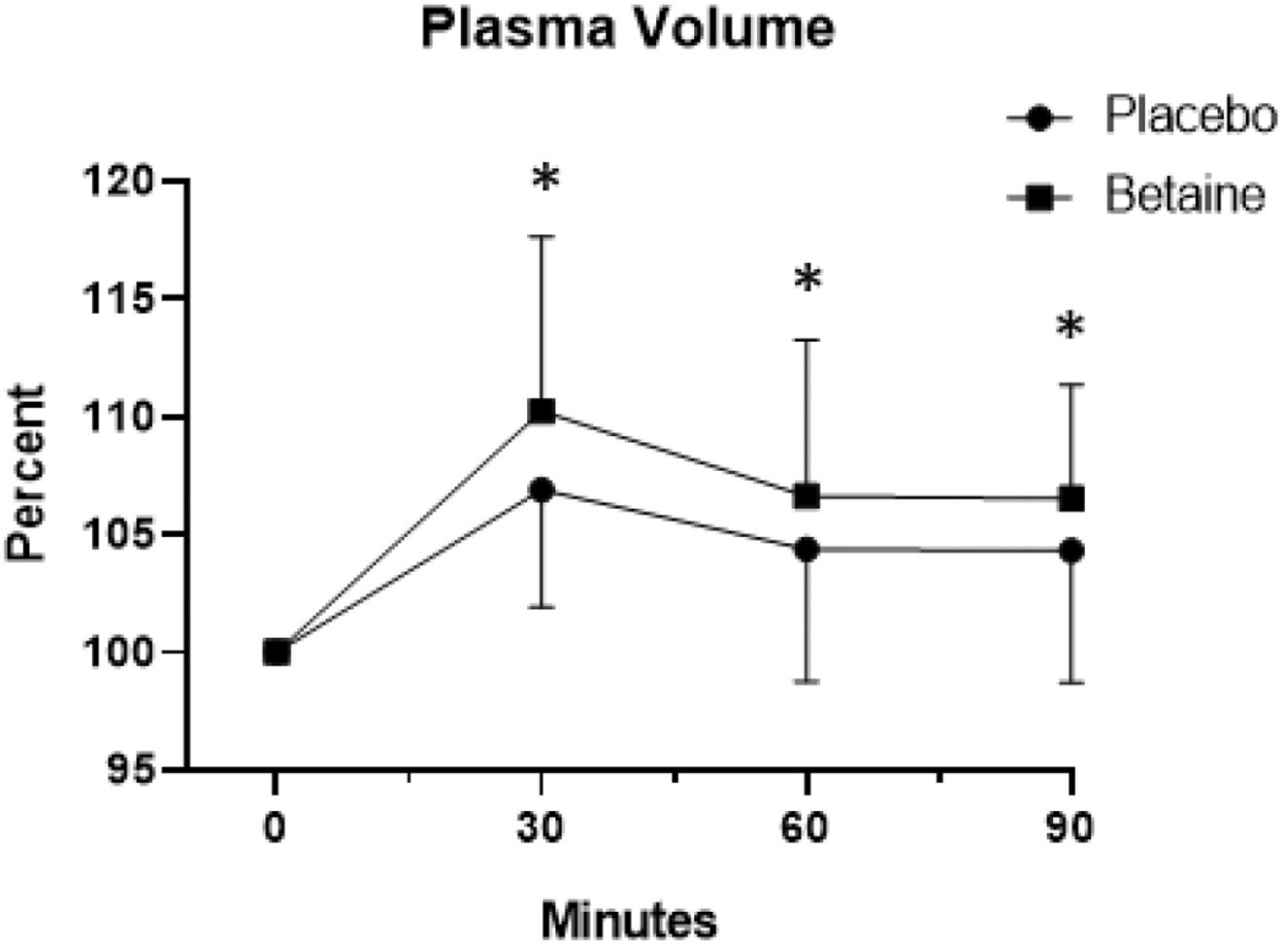
Plasma volume.

A significant time effect (*p* < 0.001), but no significant condition x time differences (*p* = 0.590) were observed for plasma volume in the heat (Table 2 & Figure 3).

Plasma osmolality could not be calculated for one individual in PLA, as there was an error regarding one time point (min 60) for plasma Na^+^. As such, the sample sizes for plasma osmolality are not identical (BET = 11, PLA = 10). A significant time effect (*p* < 0.001), but no significant condition x time differences (*p* = 0.606) were observed with calculated plasma osmolality (Table 2).

#### 3.3.3 Plasma electrolytes and proteins

The results for plasma electrolytes (Na^+^, K^+^, and Cl^−^) and plasma proteins (albumin and total plasma proteins) are shown in Table 2. Throughout the experimental trials, no significant time or condition x time effects were observed for plasma K^+^ or Cl^−^ concentrations. However, plasma Na^+^ concentrations were significantly lower in both conditions at min 30 compared to min 0 (*p* < 0.05), but returned to baseline values by min 60 and 90. Compared to min 0, plasma albumin concentrations were significantly lower at min 30 and min 60 in both conditions (*p* < 0.05), but returned to baseline values during recovery at min 90. Total protein concentrations followed a similar pattern to plasma Na+, which experienced a significant decrease by min 30 (*p* < 0.05) but returned to baseline values by min 60 and min 90.

#### 3.3.4 Sweat rate

Participants experienced a similar sweat rate under both conditions (PLA: 0.39 ± 0.16 L·h^−1^, BET: 0.37 ± 0.13 L·h^−1^; *p* = 0.742).

#### 3.3.5 Perceived thirst

**FIGURE 4:**
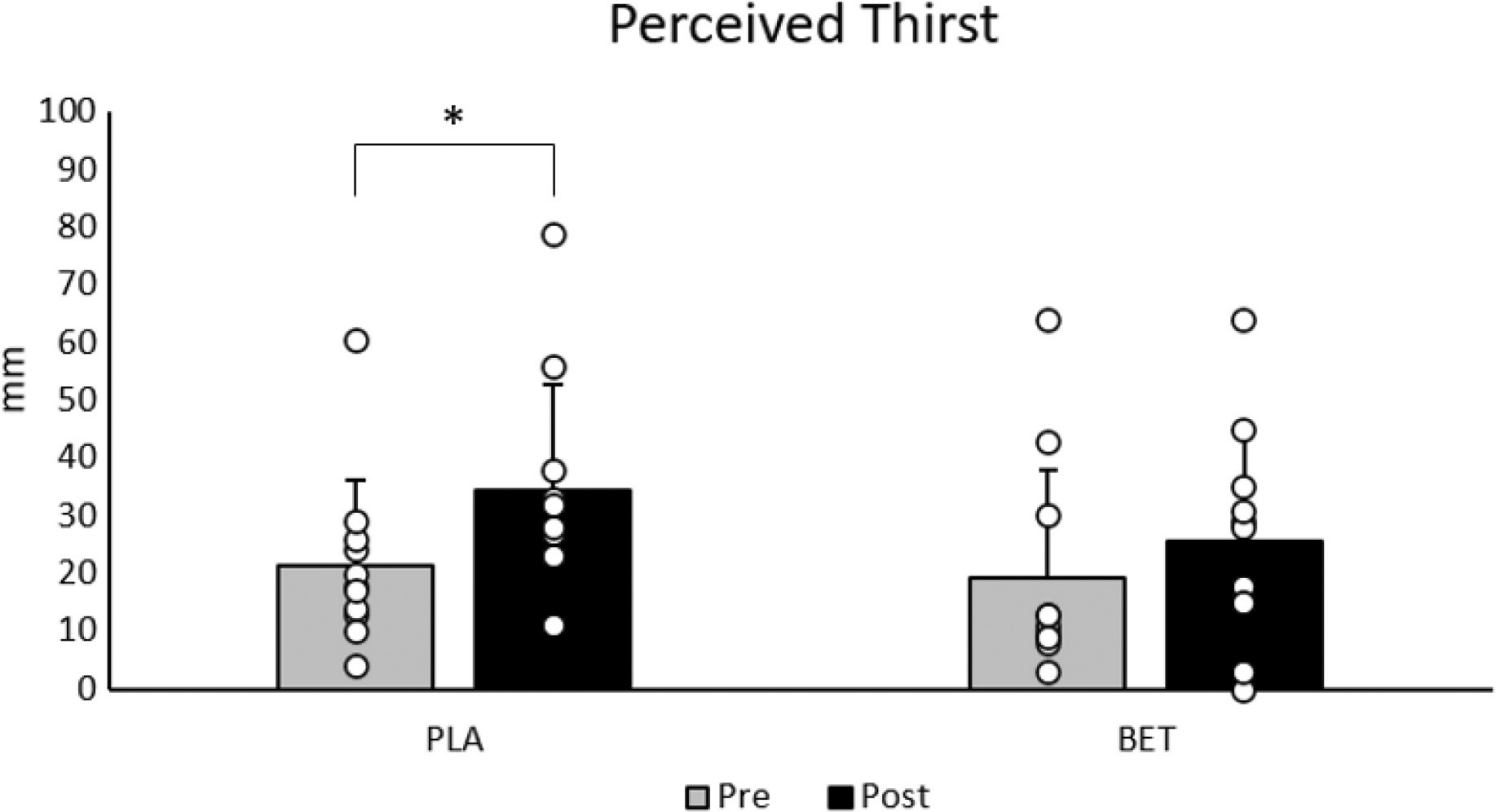
Perceived thirst.

Measures of perceived thirst immediately pre‐ versus post 60 min of passive heat exposure, were significantly increased for PLA (PRE: 21.4 ± 14.9 mm, POST: 34.4 ± 18.5 mm; *p* = 0.019), but not BET (PRE: 19.4 ± 18.7 mm, POST: 25.7 ± 18.5 mm; *p* = 0.079) (Figure 4).

### 3.4 Thermal and metabolic measures

Thermal and metabolic measures are reported in Table 3. No significant time effect (*p* = 0.392; *p* = 0.273; *p* = 0.326) or condition x time differences (*p* = 0.688; *p* = 0.161; *p* = 0.800) were observed for VO_2_, RER, or metabolic heat production, respectively throughout the experimental trials. A significant time effect (*p* < 0.001), but no condition x time differences were observed for core temperature (*p* = 0.618), skin temperature (*p* = 0.664), thermal sensation (*p* = 0.630), and HR (*p* = 0.626) throughout the experimental trial (Table 3).

**TABLE 3:** Effects of betaine on thermal and metabolic characteristics during passive heat stress.

		Min 0	Min 30	Min 60	Min 90
Core temperature (°C)	PLA	36.85 ± 0.16	36.99 ± 0.14*	37.17 ± 0.16*	37.16 ± 0.17*
	BET	36.81 ± 0.19	36.90 ± 0.22*	37.10 ± 0.31*	37.09 ± 0.23*
Skin temperature (°C)	PLA	31.45 ± 0.39	35.51 ± 0.31*	35.48 ± 0.51*	31.22 ± 1.14
	BET	31.28 ± 0.51	35.50 ± 0.28*	34.95 ± 1.84*	30.83 ± 1.35
Thermal sensation (AU)	PLA	−0.50 ± 0.87	1.82 ± 0.72*	2.77 ± 0.79*	−0.36 ± 0.50
	BET	−0.91 ± 0.94	1.91 ± 0.89*	2.64 ± 0.90*	−0.59 ± 0.66
Heart rate (bpm)	PLA	68.44 ± 9.19	78.44 ± 11.26*	85.33 ± 11.58*	67.56 ± 6.73
	BET	64.73 ± 6.44	78.18 ± 8.36*	80.27 ± 6.60*	61.18 ± 8.10
VO_2_ (L·min^−1^)	PLA	0.27 ± 0.06	0.27 ± 0.06	0.28 ± 0.05	
	BET	0.26 ± 0.05	0.27 ± 0.06	0.28 ± 0.06	
Respiratory exchange ratio	PLA	0.81 ± 0.08	0.83 ± 0.05	0.86 ± 0.08	
	BET	0.82 ± 0.06	0.81 ± 0.05	0.81 ± 0.06	
Metabolic heat production (W)	PLA	91.68 ± 19.66	90.93 ± 19.94	94.61 ± 17.72	
	BET	86.36 ± 16.26	89.14 ± 21.34	94.13 ± 20.88	

*Note*: Min 0: Immediately prior to heat exposure, Min 30: 30 min of heat exposure, Min 60: 60 min of heat exposure, Min 90: 30 min of recovery in thermoneutral environment.

Abbreviations: AU, arbitrary units, BET, betaine, BPM, beats per minute, PLA, placebo, W, watts.

* Significantly different from Min 0 (*p* < 0.05).

Core temperature was similar between conditions at rest (PLA: 36.85 ± 0.16°C, BET: 36.82 ± 0.20°C; *p* = 0.612) and both conditions experienced a similar increase after 60 min of passive heat exposure (PLA: +0.32 ± 0.22°C, BET: +0.31 ± 0.21°C; *p* = 0.912). Moreover, during the post‐heat exposure recovery portion of the trial (min 60, 75, and 90), no significant time (*p* = 0.270) or condition x time differences (*p* = 0.450) were detected. Mean skin temperature was not different between conditions at rest (PLA: 31.45 ± 0.39°C, BET: 31.28 ± 0.51°C; *p* = 0.306) or following 60 min of passive heat exposure (PLA: 35.48 ± 0.51°C, BET: 34.95 ± 1.84°C; *p* = 0.496). Moreover, during the post‐heat exposure recovery portion of the trial (min 60, 75, and 85), a significant time (*p* < 0.001) but no condition x time differences (*p* = 0.952) were detected.

### 3.5 Blood biomarkers

Plasma LPS and serum HSP70 concentrations were measured at four time points throughout each experimental condition (at rest, min 30, min 60, and min 90). A significant time effect (*p* < 0.001), but no condition x time differences (*p* = 0.183) were observed for plasma LPS. Intra‐assay coefficient of variation for LPS ELISAs were 5.85%, 7.10%, and 3.35%, respectively, with an inter‐assay coefficient of variation of 10.81%. Regarding the HSP ELISAs, two participants were below the detectable limit of the assay during one or both experimental conditions. Therefore, HSP70 data have a reduced sample size (PLA: *n* = 9, BET: *n* = 10). No significant time effect (*p* = 0.307), or condition x time differences (*p* = 0.103) were observed. Intra‐assay coefficient of variation for HSP70 ELISAs were 6.22%, 9.12%, and 9.51%, respectively, with an inter‐assay coefficient of variation of 3.63%.

## 4 DISCUSSION

The primary findings of this study were that preloading with 50 mg·kg^−1^ BET, twice per day for 7 days resulted in a significant increase in circulating BET concentrations, but did not result in significant improvements in fluid balance or heat tolerance during the loading period or during a passive heat load (60 min of sitting at 38.94 ± 0.35°C and 59.34 ± 3.27% RH) compared to PLA in this population. However, participants in PLA experienced a small, but significant increase in perceived thirst after the heat exposure, but not in BET.

### 4.1 Loading betaine

In the present study, preloading BET for 7 days did not statistically increase ICF, ECF, or TBW. These findings do not agree with those of Sayed et al. who, using an animal model, demonstrated that 500 mg·L^−1^ BET in the water supply led to improved water retention compared to the control group without BET (Sayed & Downing, 2015). Importantly, Sayed et al. also had regular exposure to high heat throughout the loading period (32°C, 80% RH, 9 h/day for 4 days). Perhaps enhanced fluid balance requires both loading BET and a thermal or hypertonic stressor presented regularly. If true, this explains why Armstrong et al., who supplemented with an acute dose of BET after the initial thermal stress, did not demonstrate improvements in hydration status during exercise, compared to water alone (Armstrong et al., 2008). Correcting for the acute dosage, the present study successfully loaded BET, yet was without a regular thermal or hypertonic stimulus during the loading period and failed to statistically increase fluid compartment volumes. Therefore, it is still unknown if loading BET while undergoing regular thermal or hypertonic stress (i.e., heat acclimation protocols) would result in greater fluid compartment volumes, compared to a placebo.

### 4.2 Betaine in the heat

#### 4.2.1 Plasma characteristics

The present BET loading protocol resulted in significant increases in plasma BET (PRE: 63.1 ± 63.3 μmol·L^−1^; POST: 702.5 ± 233.7 μmol·L^−1^, *p* < 0.001). Schwahn et al., using the same loading protocol in a similar population (i.e., healthy young men) for 5 days, report plasma BET concentrations of 1460 ± 308 μmol·L^−1^ (Schwahn et al., 2003). Armstrong et al., using an acute dose (5 g BET dissolved in 1 L water), reported peak plasma BET concentrations of 933 μmol·L^−1^ approximately 1 h after ingestion, and these values progressively decline to approximately half this concentration as the experimental trial concludes (Armstrong et al., 2008). The present data did not follow this pattern, but instead maintained a non‐significant time effect throughout the passive heat stress. This may be due to differences in fluid balance demands of active versus passive‐heat stress. Although speculative, it is possible that Armstrong et al. reported a rapid decline in plasma BET concentration because BET entered cells to help alleviate thermal and hypertonic stressors. If true, this suggests that 60 min of passive heat exposure (38.94 ± 0.35°C and 59.34 ± 3.27% RH) is an insufficient thermal or hypertonic stressor to influence these values.

#### 4.2.2 Fluid balance

Despite sweat loss, plasma volume significantly increased by 30 min in both conditions and remained elevated for the duration of the visit. The mean plasma volume increase of 10.2% at min 30 in BET, despite a net fluid loss via sweat, demonstrates the prioritization of plasma volume in the heat. As plasma volume experienced a significant mean increase (PLA: +6.9%, BET: +10.2%), there were significant corresponding dilutions in solute concentrations evident for plasma Na^+^, albumin, total proteins, and osmolality. Thus, it appears that the increased plasma volume led to decreases in solute concentration. This interpretation agrees with that of Harrison et al. who suggest the initial transient (i.e., 30–45 min) increase in plasma volume associated with passive heat exposure is a direct result of venodilation without the accompanying vasodilation (Harrison, 1986). This creates a decrease in pressure on the distal end of the capillary beds, which results in a net movement of fluid from the interstitial space to general circulation. Ultimately, there were no differences in plasma volume (*p* = 0.590) between conditions, which reveals the net movement of fluid in the short term to be similar in both groups.

#### 4.2.3 Sweat rate

In the present study, when controlling for many of the mediating factors of sweat rate (i.e., environmental conditions, clothing, hydration status, etc.), there was no difference between conditions in nude body weight and thus sweat rate during the experimental visit (PLA: −0.39 ± 0.16 L, BET: −0.37 ± 0.13 L; *p* = 0.742). These data indicate that preloading BET does not influence whole‐body sweat rate, which reinforces the findings of Armstrong et al. who also reported no changes in whole‐body sweat rate when running for 75 min at 65% VO_2_ max in the heat (31.1 ± 0.7°C, 34.7 ± 5.5% RH) (Armstrong et al., 2008).

#### 4.2.4 Subjective ratings of thirst

In the present study, perceived measures of thirst increased for PLA (PRE: 21.4 ± 14.9 mm, POST: 34.4 ± 18.5 mm; *p* = 0.019), but not BET (PRE: 19.4 ± 18.7 mm, POST: 25.7 ± 18.5 mm; *p* = 0.079) before and after the passive heat load (Figure 4). One interpretation of the present findings implies that BET attenuated a change in the thirst mechanism (i.e., plasma osmolality and plasma volume) indicating greater resilience to heat stress and resulting in a smaller magnitude of change in perceived thirst. Indeed, at min 30, plasma osmolality decreased (*p* = 0.001), with a corresponding increase in plasma volume (*p* < 0.001). While not significantly different between conditions, mean plasma volume was greater at min 30, min 60, and min 90 in BET. Likewise, despite not being significantly different, mean plasma osmolality was lower at min 60 in BET. These data may suggest that individuals in BET were able to detect acute, non‐significant changes in plasma osmolality and plasma volume associated with lower physiological strain. Thus, the visual analog scales accurately reflect an attenuated desire to drink. Our data, therefore, disagrees with that of Armstrong et al. who reported no significant changes between conditions in rating of thirst immediately post an active heat stress (Armstrong et al., 2008). However, it is important to note that the post‐heat stress values for perceived thirst were not statistically different between conditions (PLA: 34.4 ± 18.5 mm, BET: 25.7 ± 18.5 mm; *p* = 0.081). It is possible that the limitations of using visual analog scales to measure perceptions innately results in larger variations in data, minimizing the strength of this finding. As such, these findings should be interpreted with caution.

### 4.3 Thermal measures

The strongest indication of improved heat tolerance are lower core and skin temperature while experiencing the same absolute thermal load. In the present study, no significant differences were observed in core temperature between conditions at rest (*p* = 0.612), during heat exposure (*p* = 0.618) or after heat exposure (*p* = 0.450). Similarly, no significant differences were observed in skin temperature between conditions at rest (*p* = 0.306), during heat exposure (*p* = 0.664) or after heat exposure (*p* = 0.952). Together, these data do not support those found in animal models which report reduced core (Attia et al., 2009; DiGiacomo et al., 2016; Zulkifli et al., 2004) and skin (DiGiacomo et al., 2016) temperatures during and after passive heat exposure. However, these data align with those reported by Armstrong et al. (Armstrong et al., 2008). Their protocol involved participants undergoing an active 2.7% BM dehydration in the heat, acutely rehydrating with and without BET (1 L fluid ±5 g BET), then performing a strenuous run (75 min at 65% VO_2_ max) in the heat (31.1 ± 0.7°C, 34.7 ± 5.5% RH). Specifically, they reported no change in core temperature or skin temperature between conditions at min 0, 36, 72, and immediately post, the 75‐min run. Importantly, there are a few key differences in study design between the human models described presently and the animal models that demonstrated improved heat tolerance. First, Armstrong et al. examined a single dose of BET, with no loading strategy. Notably, in the animal models that demonstrate improved heat tolerance, BET is introduced chronically into feed or the water supply (DiGiacomo et al., 2016; Egbuniwe et al., 2018; Khattak et al., 2012; Ratriyanto & Mosenthin, 2018; Sahebi Ala et al., 2019; Shakeri et al., 2018; Zhang et al., 2014). However, it is possible that improved heat tolerance is only evident when chronic BET supplementation is paired with regular thermal or hypertonic stress. If true, this supports the successful animal models and explains why the human models failed to demonstrate improved heat tolerance in many physiological measures.

### 4.4 Blood biomarkers

#### 4.4.1 Lipopolysaccharide

In the present study, the passive heat load did not increase core temperature to a point where damage to the gut would be suspected (37.13 vs. 39.0°C) (Pires et al., 2017). In fact, the significant time effect (*p* < 0.001) revealed mean plasma LPS decreasing in both conditions by min 30 and remaining decreased through min 90. Although not directly measured, it could be that increased cardiac output in the present study, via the observed increase in heart rate (*p* < 0.001) and cutaneous blood flow as evidenced by increased mean skin temperature (*p* < 0.001), enhanced clearance rates and is responsible for decreases in plasma LPS concentration.

Elevated core temperature (> 39.0°C) has been shown to reliably induce LPS endotoxemia in a healthy GI tract (Pires et al., 2017). However, it appears to be accelerated by mechanical (repeated movements through exercise) and chemical (hypoxia secondary to visceral vasoconstriction) disruptions to the enterocytes (Yeh et al., 2013). Yeh et. al, examined LPS concentrations in 15 (14 men, one woman) fit (48.9 ± 3.0 mL·kg·min^−1^) participants running for 60 min at 70% VO_2_ max on a treadmill in the heat (33°C, 50% RH) or cool (25°C, 60% RH) (Yeh et al., 2013). Despite the constant mechanical disruption in both conditions, only those in the hot condition had significantly greater plasma LPS concentrations immediately after the run. In the present study, the passive nature of the heat stress lacked mechanical disruptions and failed to surpass the core temperature threshold needed to demonstrate LPS endotoxemia. Future research should examine BET's potential impact on enterocyte integrity and LPS endotoxemia during active heat stress when core temperatures cross the threshold and mechanical disruption is present.

#### 4.4.2 Heat shock protein 70

In the present study, no significant time effect (*p* = 0.307), or condition x time differences (*p* = 0.103) were observed for HSP70. Out of the 11 participants, two were below the detectable limit of the assay during one or both experimental conditions (PLA: *n* = 9, BET: *n* = 10). Together with the relatively small magnitude of change evidenced in other physiological markers for heat tolerance in the present study (i.e., core and skin temperature, metabolic heat production, sweat rate, etc.), it becomes clear that the passive heat in the present study was not great enough to elicit a change in serum HSP70. As such, these data cannot satisfy the question of BET's ability to promote heat tolerance through reduced demand for HSP expression. Future research with greater thermal stressors (i.e., exercise to promote increases in metabolic heat production) is warranted.

### 4.5 Metabolic variables

Despite similar core and skin temperatures between conditions in the present study, BET has been reported to impact metabolism in the heat (Armstrong et al., 2008; Cholewa et al., 2018; Huang et al., 2015; Sahebi Ala et al., 2019) and thereby alter metabolic heat production. Metabolic heat production can be estimated by changes in heart rate and can be calculated directly with measures of VO_2_ and RER (Cramer & Jay, 2016; Murgatroyd et al., 1993). In the present study, no significant differences were observed between conditions for heart rate (*p* = 0.626), VO_2_ (*p* = 0.688), or RER (*p* = 0.161) at any timepoint. Calculated metabolic heat production, also did not change over time (*p* = 0.326) or between conditions (*p* = 0.800). Together, these data demonstrate that preloading BET does not alter whole‐body metabolism and thereby metabolic heat production during a passive heat load.

### 4.6 Limitations

When examining the previous successful thermoregulatory results demonstrated in animal models, it becomes clear that loading the supplement regularly is a key element to elicit the desired changes (Willingham et al., 2020). However, despite preloading BET in the present study, no physiological biomarkers in the heat were enhanced due to BET. Despite falling in the uncompensable range established by Vecellio et. al, during minimal activities of daily living (Vecellio et al., 2022), it is possible that the thermal load induced by passive heat stress was insufficient in magnitude (i.e., intensity and duration). However, we deemed this a necessary first step to match most of the passive heat literature in animal studies and use as a steppingstone for future work with more extreme conditions. Nevertheless, the intensity (40°C, 60% RH) in the present study closely parallels previous successful passive heat load models and is therefore unlikely to be the limiting factor (DiGiacomo et al., 2016; Park & Kim, 2016; Sahebi Ala et al., 2019; Zulkifli et al., 2004). Thus, if magnitude is the limiting factor, it is more likely to be a function of duration. Indeed, successful animal model interventions use 4 h (Zulkifli et al., 2004), 6 h (Attia et al., 2009; Dangi et al., 2016), and even as high as 8 h (DiGiacomo et al., 2016); (Park & Kim, 2016) per day of a similar thermal load to the present study. Alternatively, the limiting factor may be a function of frequency. In the present study, participants experienced one bout of heat exposure. Whereas, successful animal models intervene with regular, cyclical heat stress for many days in a row.

Further, this study was designed to determine if the known impacts of BET supplementation would translate from lower order models (i.e., cell culture, plant, and animal models) to humans. As such, to minimize the known impacts of fluid (White et al., 2011) and temperature (Kawamori et al., 2019) shifts associated with the menstrual cycle, as well as known differences in methylation reactions between genders (Xu et al., 2008) women were excluded. Therefore, our data are specific to young recreationally active men.

## 5 CONCLUSIONS AND FUTURE RESEARCH

Collectively, these data do not support the hypotheses that BET improves fluid balance and heat tolerance in humans undergoing 1 h of passive thermal stress in young, healthy men. Future research should examine the impact of daily BET supplementation on individuals undergoing active heat stress, with a sufficiently great internal and external thermal load to elicit core temperatures beyond 39.0°C, to determine if BET can promote GI integrity and thereby attenuate or prevent endotoxemia during exercise in the heat.

## AUTHOR CONTRIBUTIONS

Conceptualization, B.D.W. and M.J.O; data curation, B.D.W., L.I.R., and T.J.R.; writing—original and draft preparation, B.D.W., L.I.R., and T.J.R.; writing—review and editing, B.D.W., L.I.R., T.J.R., and M.J.O.

## FUNDING INFORMATION

This study was funded by NOW Foods, Inc. under Grant RF04017. MJO serves on the scientific advisory board for the Korey Stringer Institute.

## CONFLICT OF INTEREST STATEMENT

All other authors have no competing interests to declare.

## ETHICS STATEMENT

This study was conducted according to the guidelines laid down in the Declaration of Helsinki and all procedures involving human subjects were approved by the Florida State University Institutional Review Board [00000259].
